# The performance costs of interruption during visual search are determined by the type of search task

**DOI:** 10.1186/s41235-021-00322-0

**Published:** 2021-08-20

**Authors:** David Alonso, Mark Lavelle, Trafton Drew

**Affiliations:** Department of Psychology, University of Utah, Salt Lake City, UT USA

**Keywords:** Interruptions, Visual search, Medical image perception, Working memory

## Abstract

Prior research has shown that interruptions lead to a variety of performance costs. However, these costs are heterogenous and poorly understood. Under some circumstances, interruptions lead to large decreases in accuracy on the primary task, whereas in others task duration increases, but task accuracy is unaffected. Presently, the underlying cause of these costs is unclear. The Memory for Goals model suggests that interruptions interfere with the ability to represent the current goal of the primary task. Here, we test the idea that working memory (WM) may play a critical role in representing the current goal and thus may underlie the observed costs associated with interruption. In two experiments, we utilized laboratory-based visual search tasks, which differed in their WM demands, in order to assess how this difference influenced the observed interruption costs. Interruptions led to more severe performance costs when the target of the search changed on each trial. When the search target was consistent across trials, the cost of interruption was greatly reduced. This suggests that the WM demands associated with the primary task play an important role in determining the performance costs of interruption. Our findings suggest that it is important for research to consider the cognitive processes a task engages in order to predict the nature of the adverse effects of interruption in applied settings such as radiology.

## Significance

Tasks, such as medical evaluations within diagnostic radiology, vary in their complexity and design. Interruptions that occur during the evaluation of medical images may reduce a radiologist’s ability to diagnose disease efficiently, accurately, or both. Our research suggests that the primary task may help determine the nature of the associated interruption cost. Thus, we may be able to reduce the frequency of medical errors in diagnostic radiology by identifying the circumstances in which interruptions are less likely to lead to errors. In our study, we demonstrate that the design of the primary task modulates the adverse effects of interruptions on human performance. Across two experiments, we show that interruptions lead to larger performance costs when a visual search task requires an observer to find new information, rather than the same information from trial-to-trial. These results suggest that the memory demand associated with the primary task is a critical factor in determining whether an interruption will lead to more errors or simply increase the time it takes to reach the same decision.

## Introduction

Recall a moment when your conversation with a friend was interrupted. Upon resuming the conversation, there may have been difficulty remembering the topic of the conversation. Now, imagine being interrupted during a moment when there are life and death consequences, for example: radiologists who search through medical images for cancer or TSA agents who screen baggage for potentially harmful objects. Here, an interruption may be more than just a nuisance. Instead, it may lead to serious errors. Therefore, our current research aims to determine whether interruption leads to decrements in performance during visual search and the circumstances that exacerbate these performance decrements. Furthermore, our focus on visual search is motivated by the possibility that we can identify factors that hinder radiologists’ ability to search through medical images and diagnose disease.

In a 2000 report, the Institute of Medicine reviewed the problems related to medical errors, what contributes to these errors, and what can be done to reduce their frequency. In this review, medical errors emerged as one of the leading causes of deaths in America (Kohn et al., 2000). Furthermore, this review asserted that certain work conditions may increase the likelihood for errors to occur if systems are designed in such a way that does not take into account the limits of human performance. We argue that the work environment of radiologists is one such system. Studies have quantified how often an on-call radiologist experiences interruption during busy work hours and has revealed alarming results in which a radiologist may be interrupted on average 2.5 times during the evaluation of a CT scan (Yu et al., 2014).

Among many other responsibilities, a radiologist’s primary role involves the careful viewing and evaluation of complex medical images. Interruptions that occur during medical image evaluations may set the stage for an environment in which costly mistakes are more likely to occur. This possibility becomes more urgent to consider because research has identified a link between interruptions and an increased rate of clinical errors that occur during the administration of medicine (Westbrook et al., 2010). This prior research has found that interruptions lead to a variety of negative outcomes in a clinical setting, including increased rates of incorrect drug prescriptions (Westbrook et al., 2010) and increased rates of disagreements between resident and attending radiologists (Balint et al., 2014). Notably, the prior work with radiologists provides indirect evidence that interruptions lead to negative outcomes, finding: that epochs of time with more telephone calls were associated with an increase in discrepant medical reports (Balint et al., 2014).

Research in cognitive psychology strongly suggests that interruption is a prime candidate for understanding the work conditions that can lead to poor performance. A great deal of evidence indicates that interruptions are disruptive to primary task performance. The disruptive effects of interruption typically lead to an increase in the time it takes to complete a primary task and an increase in the amount of errors made during the primary task (Altmann et al., 2014; Li et al., 2008; Monk et al., 2008; Trafton et al., 2003, 2011; Zish et al., 2015). These accuracy and time costs are typically observed during computer-based procedural tasks with predefined steps that must be completed in a specific sequence. For example, the UNRAVEL task is often used because it consists of multiple subtasks (i.e., steps), and interruptions have been shown to interfere with peoples’ ability to perform the subtasks in a timely manner, in the correct order, or both. UNRAVEL and other similar procedural tasks have provided valuable ways to measure the disruptive effects of interruption and its association with performance decrements. The current literature has focused on these tasks because they lend themselves to examining models that describe how goals are suspended and resumed. In addition, these tasks also provide data that can be analyzed for sequence errors such as repeating or skipping a certain action in a sequential task. For example, Altmann and Trafton’s (2002) Memory for Goals (MFG) has been successful in guiding theoretically grounded research on how interruptions may disrupt performance during a primary task. The core principle underlying this model is that we store memories that represent our goals during a given task. Furthermore, when multiple memories are stored that pertain to different goals, the memory with the highest activation is most effectively recalled following an interruption. In the MFG model, the memory with the highest activation is determined by how recently it was stored and how frequently it has been used. In this context, interruptions force goals to be suspended thereby challenging our ability to store and retrieve the memories that represent our goals. Goals within the MFG model are temporary subgoals that guide performance on sequential tasks, such as UNRAVEL, by serving as markers that indicate whether an action needs to be completed or has been finished.

Predominant models of working memory (WM) suggest that the function of WM is to provide temporary storage of information that can be retrieved in order to carry out cognitive tasks (Cowan, 1998). In this respect, WM seems like a likely center for the storage of temporary subgoals that change on a moment-to-moment basis depending on the subtask at hand. Moreover, research has shown that WM resources are relied upon during the pursuit and achievement of goals that revolve around cognitive tasks (Avery et al., 2013). This suggests that there is a link between the MFG model of interruption and WM, such that interruptions may disrupt the ability to maintain task-relevant information in mind.

Due to the focus on understanding sequence errors and how interruption can give rise to these errors, few studies have examined how interruptions may impact tasks with a single goal that extends through time, such as visual search. In contrast to a sequential task with predefined steps in a specific order, visual search in a medical context often involves a single task (e.g., find signs of cancer) rather than a series of unique tasks that must be completed in order. Therefore, the design of visual search tasks differs in important ways from the procedural tasks that have been used to study the impact of interruptions on behavior. Thus, it is unclear to what extent the predictions regarding the effects of interruption will apply to visual search behavior. In some cases, brief and cyclic interruptions seem to increase the speed at which people resume a visual search task (Lleras et al., 2005). However, the interruptions in this research involved interspersed replacements of the search array with a blank screen for short durations (900–3400 ms) in order to test the role of memory during visual search. The interruptions did not involve the need to complete a secondary task, rendering it less representative of the type of interruptions that occur in more naturalistic settings. In particular, our laboratory became interested in studying the costs of interruption after radiologist collaborators frequently cited telephone interruptions as one of the most difficult part of their job. In order to try to understand this problem better, Williams and Drew (2017) evaluated the disruptive effects of abrupt, unpredictable interruptions that involved a secondary task during visual search. This study utilized a visual search task in which undergraduates searched through volumetric chest CT scans for small lung nodules. Participants were asked to emulate the search behavior of a radiologist. Overall, their investigation found that interruptions led to a reliable time cost but no effect on accuracy on primary task performance. Eye tracking measures also revealed that interruption disrupted the ability to remember previously examined regions of the lungs prior to the interruption.

However, an important aspect of visual search that was not explored in this prior study involves the concept of target templates. A target template is a representation of an object that is held in memory and guides attention during visual search (Bundesen, 1990; Bundesen et al., 2005; Desimone & Duncan, 1995; Duncan & Humphreys, 1989; Woodman et al., 2007). In the context of visual search, electrophysiological research suggests that when targets are new from trial-to-trial, WM is recruited to update and store the new target template (Carlisle et al., 2011; Woodman & Arita, 2011). Whereas when targets repeat from trial-to-trial, the target template is handed off to long-term memory (LTM) (Carlisle et al., 2011; Woodman et al., 2013). In Williams and Drew’s (2017) study, participants searched for simulated lung nodules that remained the same throughout the study. Therefore, the target template that guided participants’ search did not change or heavily rely on WM resources involved in the storage and maintenance of target templates.

The Williams and Drew (2017) study was an important first step in quantifying the effects of interruption on visual search. However, since the variability of target templates was not explored in this study, we do not know how interruptions may impact visual search when target templates change from trial-to-trial or when they remain the same. Exploring this issue is important because the disruptive effects of interruption during visual search may largely depend on the variability of target templates and the different demands they place on WM. Consistent with this possibility, Kunar et al. (2017) found that during search for cancer in mammograms, there was an increase in false alarms and more targets were missed when there was a range of potentially cancerous masses to search for across trials compared to a specific type of cancer. This finding was explained in the context of research which theorizes that searching for multiple targets limits the ability to store high quality representations of targets in WM, compared to searching for a single well-defined target (Kunar et al., 2017). Though this study provided evidence that is consistent with this proposal, it is unknown how manipulating the quality of the target representation (and the subsequent WM demands) will interact with interruptions.

There is also evidence to suggest that WM plays a critical role during interruptions (Drews & Musters, 2015; Foroughi et al., 2016; Meys & Sanderson, 2013; Werner et al., 2011). Research in this domain has typically focused on comparing the effects of interruption between individuals with high and low WM capacity. This research has found that people with greater WM capacity are able to better handle the disruptive effects of interruption during procedural tasks (Drews & Musters, 2015; Foroughi et al., 2016; Meys & Sanderson, 2013; Werner et al., 2011). Yet, this research still leaves an open question related to how interruptions may influence behavior when cognitive tasks place different demands on WM. By addressing this question, we hope to shed light on two areas of research. First, we can test the extent to which the disruptive effects of interruption generalize to visual search tasks that differ in their cognitive demands. By doing so, we can provide critical information to guide future research with expert populations such as radiologists. Second, we can gain further insight into the role WM plays when tasks are interrupted. This second issue is highly pertinent to the existing literature because interruptions are believed to exert their disruptive effects by interfering with memory (Altmann & Trafton, 2002; Trafton et al., 2011).

In the current study, we aimed to determine if the cost of interruption depends on whether the search target remained the same throughout the experiment or changed on each trial. Consequently, the current work is designed to investigate how interruptions impact search performance when target templates change, thereby theoretically recruiting more WM resources. We predicted there would be a larger cost of interruption when search involved looking for new targets than when the target was consistent throughout the experiment. When the target changed on each trial, we expected that WM would maintain target templates during search and that interruptions would disrupt this process. In contrast, when searching repeatedly for the same target, we predicted target templates would be stored in LTM and resistant to the disruptive effects of interruptions. Ultimately, the goal of our work is to identify the factors that modulate the cost of interruption during visual search (i.e., the memory demands associated with the primary task) in order to inform future research with radiologists.

## Methods

### Experimental design and primary task

Our experiments were hosted on Pavlovia.org to permit online data collection from the recruitment service Prolific.co. All experiments were developed using PsychoPy (Peirce et al., 2019) and adapted to jsPsych (de Leeuw, 2015). Stimulus presentation was assessed in the laboratory on a 20’ Asus flat screen monitor that was positioned 70 cm away from participants. Under these conditions, stimuli subtended approximately 1.5° of visual angle. Because we could not control the size of participants’ computers and their seating distances, we cannot report the exact size of stimuli as they appeared to participants.

For stimuli, we generated twenty different sets of 310 search arrays with an identical copy for Experiment 1 and Experiment 2. The only difference between the two copies is that the target was different within a set in Experiment 1, but in Experiment 2, the target remained the same within a set. In other words, there was a new target on every trial in Experiment 1, but in Experiment 2, the target was the same across trials. Each set contained 10 practice trials and 300 experimental trials that were divided into two blocks (150 trials in each block). Trials were broken up into 50% target-present trials where a single target was visible and 50% target-absent trials. Within practice and experimental trials, the order of the search arrays was randomized for each person. Each visual array contained 150 objects from the Brady et al. (2008) memory database. On target-present trials, the target appeared in the 15 center-most grid-positions 10% as frequently as would be expected by chance to reduce the probability that participants would see the target immediately after the appearance of the search array. Objects were otherwise randomly placed on an equally spaced 17 × 9 grid with random jitter such that stimuli could slightly overlap. When the target overlapped with a distractor, it was always in front of the distractor so that the target was never occluded. Blank space subtending 1.2° padded the search array from above and below, and 1.1° padded the right side. On the left side, 0.5° of blank space separated the “absent” box from the search array, and an additional 0.5° separated the “absent” box from the screen’s edge. We used a large visual set size to increase the time it took to find the target for two reasons. First, we wanted to increase the likelihood that interruptions occurred before search was completed. Second, we wanted the amount of time that participants spent searching to be more comparable to the amount of time radiologists spend on a medical evaluation. For example, in prior work, our group found that radiologists examined mammograms for signs of breast cancer for ~ 100 s on each case (Drew and Musters, 2015).

**Fig. 1:**
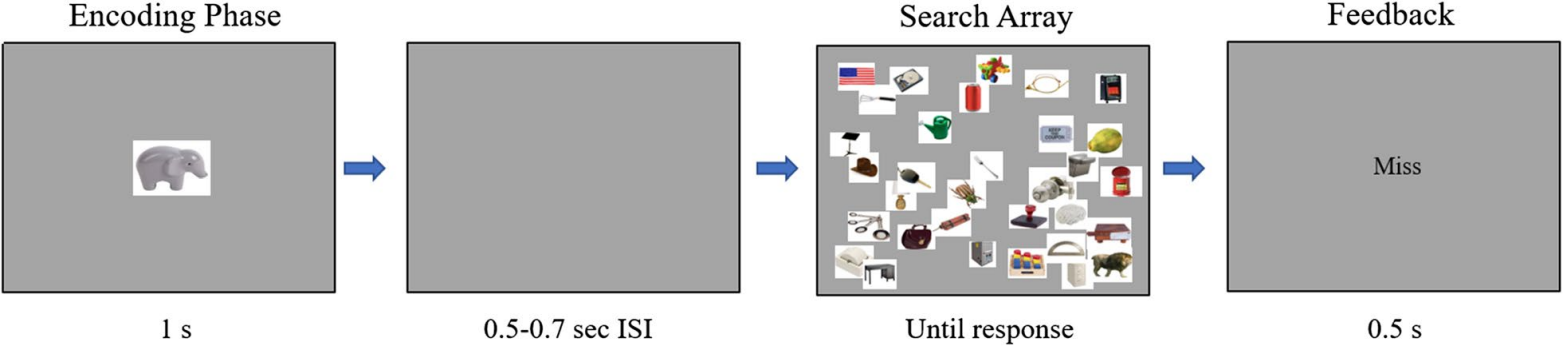
Layout of an uninterrupted trial. During uninterrupted trials, search was not suspended by the interruption task. For demonstration purposes, the schematics throughout the paper contain less than 150 distractors and do not show the “absent box”

Each trial began with a preview of the target (1 s) presented at the center of the screen. Following the preview of the target item, a blank gray screen appeared, followed 0.5–0.7 s later by a visual search array with 150 objects. Participants were instructed to click on the target if it was present. If the target was absent, they were to click on a designated area to the left of the search array that was the same size as the stimuli (the “absent” box). A click near the appropriate location was considered correct if it landed in a virtual square that was twice as wide and twice as high as the stimuli, centered on the appropriate location. The search array remained on the screen until a response was made. After a response, performance feedback was presented on the center of the screen for 0.5 s indicating to participants whether they identified the target (‘hit”), missed the target (“miss”), correctly determined that the target was absent (“correct rejection”), or incorrectly determined the target was present (“false-alarm”). Participants initiated the next trial at their own pace with a keyboard press (Fig. 1).

### Interruption task

**Fig. 2:**
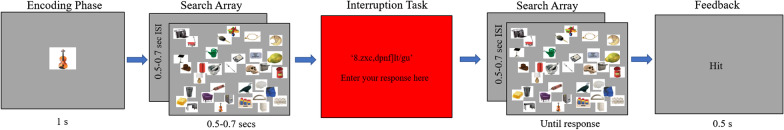
Layout of an interrupted trial. In Experiment 1, participants searched for a new target on every trial. In Experiment 2, participants searched for the same target on each trial. Search arrays contained 150 distractors. Interruptions occurred 0.5–0.7 s from the onset of the search array

In both experiments, throughout the course of the search task, participants were interrupted on 10% of trials and required to complete a secondary task. The interruption always occurred while the search array was on the screen. Interruptions began shortly after search onset in order to minimize the likelihood that a participant would finish their visual search before being interrupted. In addition, the task was designed so that participants were able to begin searching (i.e., saccade at least twice) before being interrupted. Reaction times from pilot data were slower than 0.6 s on 97% of trials. Therefore, the search array was replaced by the interruption task after a delay of 0.5–0.7 s. In total, participants were interrupted 30 times during the experimental blocks (15 interruptions during the first block and 15 interruptions during the second block) and two times during the practice trials. An equal number of target-present and target-absent trials were interrupted. In addition, interruptions were designed to randomly occur within a block with the constraint that interruptions never occurred on consecutive trials. Interrupted trials followed a similar format as uninterrupted trials with a few key differences. On interrupted trials, the visual array was replaced with a red screen, which contained all the relevant information for the secondary task. Participants were presented, in the center of the screen, a series of random characters (e.g., “8.zcx,dpnf]lt/gu”) which they typed into the computer. In order to complete the interruption task and resume search, participants were required to correctly type 80% of the random characters. Once the information was typed, participants indicated that they had completed the interruption task via a keystroke. At this point, a blank gray screen appeared for a random interval between 0.5 and 0.7 s and the search array returned to the screen. The series of random characters for the interruption task were created prior to the experiment and were different on every interruption. Characters were chosen to form a random combination of letters, numbers, and symbols. The interruption task was the same for both experiments (Fig. 2).

### Sample size

Power calculations for the current study were based on data that were collected from a pilot study that followed a similar design. In the pilot study, we compared search accuracy between trial-type (uninterrupted and interrupted) and search-type (search for new targets or search for the same target). Trial-type was a within-subjects factor, and search-type was a between-subjects factor. Analyses on the pilot data provided a repeated measures correlation of 0.43 between uninterrupted and interrupted accuracy. We found a significant interaction between trial-type and search-type on search accuracy: Cohen’s *f* = 0.33. Unfortunately, these prior experiments are difficult to interpret because the interruptions were beeps that were played through a phone and initiated by an experimenter. Although this design is closer to our interest in interruptions in the radiology reading room, the design did not control for the timing of the interruptions such that interruptions may have systematically occurred on more difficult visual search trials. This made it difficult to interpret these results and ultimately led to the current investigation. Based on the parameters from our pilot study and *α* = 0.05, we determined that we needed a total sample size of 36 (18 participants in each experiment) in order to achieve 95% power and detect a similar effect for the current study.

This is the first interruption experiment our laboratory has conducted online. Thus, we exceeded the recommended sample size from our power calculations in order to assess the quality of data collected from online experiments. With this in mind, we collected data from 31 good subjects in each experiment (total *N* = 62). This provided us with valuable information for future online studies, such as the number of participants that complete the current experiments with reasonable performance. This is important because online studies typically involve brief (30 min) tasks and our study was estimated to take 1.5 h. Overshooting the sample size also addressed the possibility that the effect sizes for the current study are reduced by participants that complete these experiments in uncontrolled environments, rather than a laboratory. Our sample size justification, hypotheses and primary planned analyses were pre-registered at OSF (https://osf.io/4v3kp).

### Participants

All experiments and materials were approved by the University of Utah Institutional Review Board. Participants enrolled for the study via Prolific.co and were compensated $11.04 for completing the experiment. The median duration to complete the study from reading the consent form to completing follow-up questions after the end of the experiment was 1.5 h. These questions asked if the participants noticed problems with the experiment, where they completed the study, and whether they were interrupted by their surroundings during the study. The processing speed of participants’ computers was tested during the practice trials. If the requested durations (e.g., 1000 ms for the target preview) were prolonged by more than 100 ms or if the variability in delays exceeded 100 ms, then the session was terminated and the participant was paid a prorated amount. In Experiment 1, 33 participants participated after providing informed consent. Data from two participants were discarded; one participant did not complete the study and the other participant’s search accuracy was below 60%. Analyses were conducted on 31 participants (16 women, 15 men, mean age = 30.1, age range = 21–55). For Experiment 2, 32 participants participated after providing informed consent. Data were discarded from one participant due to an unknown technological failure where trials for this individual failed to report timing information. For Experiment 2, analyses were conducted on 31 participants (14 women, 17 men, mean age = 34, age range = 21–58). From the time of consent to the last experimental trial, on average, Experiment 1 was completed 1.3 h and Experiment 2 in 1.1 h.

## Results

### Interruption task

On average, the interruption task was completed in 20.6 s (SD = 14.3 s) in Experiment 1 and 17.1 s (SD = 5 s) in Experiment 2. The average duration of the interruption task did not significantly differ between experiments (*t*(60) = 1.38, *p* = 0.17, Cohen’s *d* = 0.35). It is important to note that the design of our study did not entirely preclude participants from indicating whether their target was present or absent before the onset of the interruption task. However, in our analyses, we checked for this and determined that it did not occur in either Experiment 1 or 2. The interruption task was not further analyzed.

### Primary task

Response times that were 200 ms or 3 SD above from a participant’s mean on present or absent trials were removed from the analyses. In Experiment 1, this led to an average of 0.64% interrupted trials and 5.5% uninterrupted trials that were excluded from the analyses. In Experiment 2, this led to an average of 0.93% interrupted trials and 8.7% uninterrupted trials that were excluded from the analyses. We conducted a mixed-effect ANOVA in order to evaluate the effect of interruption on search accuracy between Experiment 1 and 2. When comparing performance between Experiment 1 and 2, trial-type (uninterrupted and interrupted) and search-type (Exp. 1: search for new targets, or Exp. 2: search repeatedly for the same target) were included as the two factors of interest. We hypothesized a significant interaction between trial-type and search-type such that the difference in search accuracy between interrupted and uninterrupted trials was larger in Experiment 1. We conducted a similar mixed-effect ANOVA in order to examine the effect of interruption on RTs. However, we did not expect a significant interaction between trial-type and search-type. Rather, we predicted that there would be a main effect of trial-type where interruptions led to a similar increase in RTs during both experiments. For the planned comparisons, we conducted a set of one-tailed paired sample *t* tests where we compared search accuracy and RTs between uninterrupted and interrupted trials in Experiment 1 and 2, respectively. Our RT analyses were conducted on trials with correct responses. All our analyses are in accordance with our pre-registration on the Open Science Framework.

**Fig. 3:**
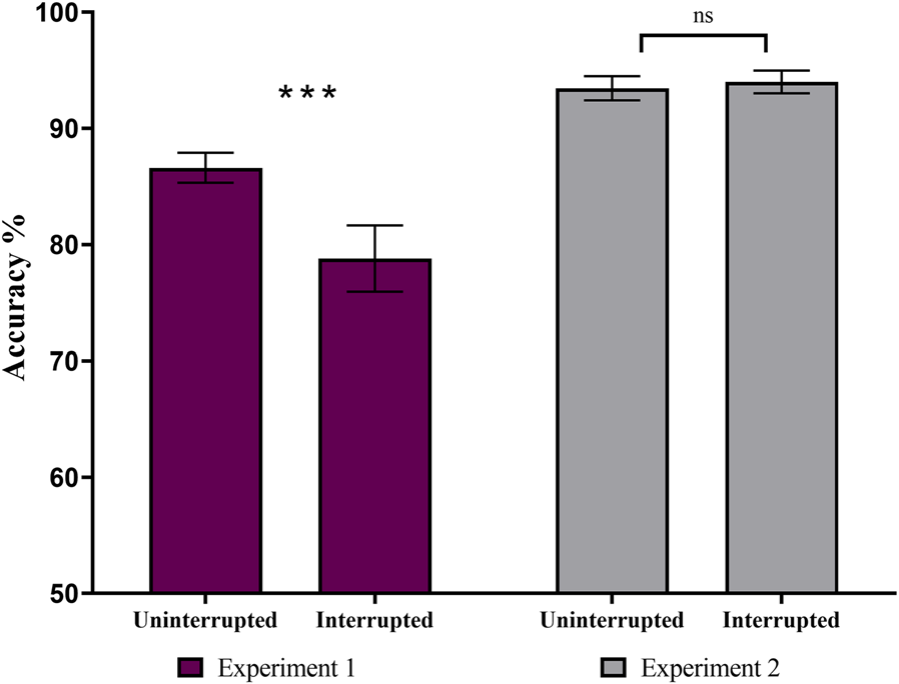
We observed a significant interaction between trial-type (uninterrupted, interrupted) and search-type (Exp. 1: search for new targets, or Exp. 2: search repeatedly for the same target) on primary task accuracy. Accuracy was significantly lower during interrupted trials in Experiment 1 but not during Experiment 2. The stars denote significance (*p < .05, **p < .01, ***p < .001). The error bars represent the standards error of the mean

The mixed-effects ANOVA on search accuracy showed a significant main effect of trial-type [*F*(1,60) = 10.30, *p* < 0.01, *η*^2^ = 0.03] and search-type [*F*(1,60) = 26.13, *p* < 0.001, *η*^2^ = 0.25]. The interaction between trial-type and search-type on search accuracy was also significant [*F*(1,60) = 13.62, *p* < 0.001, *η*^2^ = 0.04, see Fig. 3]. In terms of the main effect of search-type, search accuracy was higher overall in Experiment 2 compared to Experiment 1. For the significant interaction, planned comparisons revealed a significant difference in search accuracy between interrupted and uninterrupted trials in Experiment 1 (interrupted: *M* = 78.7%, SD = 15.9%, uninterrupted: *M* = 86.5%, SD = 7.1%, *t*(30) = 3.83, *p* < 0.001, Cohen’s *d* = 0.68, see Fig. 3). However, in Experiment 2, the difference in search accuracy between interrupted and uninterrupted trials was not significant (interrupted: *M* = 93.9%, SD = 5.4%, uninterrupted: *M* = 93.4%, SD = 5.7%, *t*(30) = − 0.55, *p* = 0.70, Cohen’s *d* = − 0.09, see Fig. 3). Thus, the significant difference in search accuracy between interrupted and uninterrupted trials in Experiment 1 appears to be driving the observed significant interaction between trial-type and search-type.

**Fig. 4:**
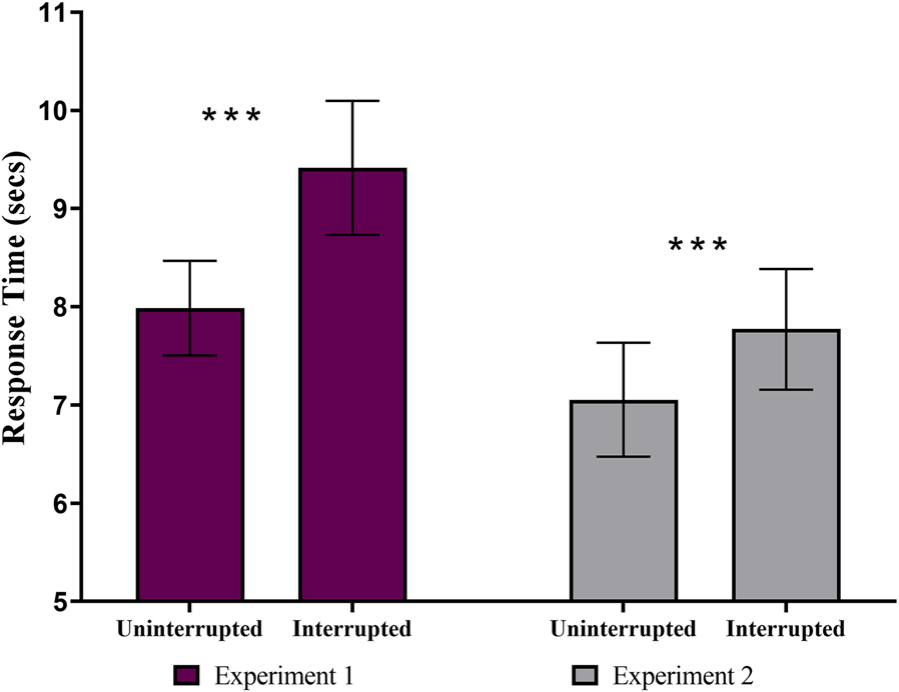
There was a significant main effect of trial-type (uninterrupted, interrupted) on primary task RTs. The main effect of search-type (Exp. 1: search for different targets, or Exp. 2: search repeatedly for the same target) was not significant. The interaction between trial-type and search-type was significant. Critically, RTs were significantly slower during interrupted trials in Experiment 1 and Experiment 2. The stars denote significance (*p < .05, **p < .01, ***p < .001).The error bars represent the standard error of the mean

In order to determine whether interruptions led to slower RTs, we first controlled for the duration of the interruption task by subtracting it from RTs on interrupted trials. After this correction, the mixed-effects ANOVA on RTs revealed a significant main effect of trial-type on RTs [*F*(1,60) = 38.96, *p* < 0.001, *η*^2^ = 0.02]. However, the main effect of search-type on RTs was not significant [*F*(1,60) = 2.46, *p* = 0.12, *η*^2^ = 0.03]. The interaction between trial-type and search-type on RTs was significant [*F*(1,60) = 4.2, *p* = 0.04, *η*^2^ = 0.002]. In terms of the main effect of trial-type, planned comparisons showed that RTs were significantly higher during interrupted compared to uninterrupted trial in Experiment 1 (interrupted: *M* = 9.4 s, SD = 3.7 s, uninterrupted: *M* = 7.9 s, SD = 2.6 s, *t*(30) = − 4.36, *p* < 0.001, Cohen’s *d* = − 0.78, see Fig. 4) and Experiment 2 (interrupted: *M* = 7.7 s, SD = 3.4 s, uninterrupted: *M* = 7.0 s, SD = 3.2 s, *t*(30) = − 6.73, *p* < 0.001, Cohen’s *d* = − 1.2, see Fig. 4). The significant interaction between trial-type and search-type indicates that the effect of interruption on RTs depended on whether search consisted of finding new targets or repeatedly searching for the same target.

## Discussion

The primary goal of this investigation was to explore the effects of interruption on visual search. We aimed to determine how the costs of interruption would differ when search consisted of looking for a new target on each trial (Experiment 1) versus targets that were consistent throughout the experiment (Experiments 2). Prior research suggests that in Experiment 1, successful search depended on the recruitment of WM resources for the storage and maintenance of target templates, whereas in Experiment 2, target representation was held in a more durable format, such as LTM (Carlisle et al., 2011; Woodmanet al., 2013). Consistent with this proposition, across our two experiments, we observed an interaction between interruption and the type of search participants engaged in on search accuracy. The interaction was driven by a significant difference in accuracy between uninterrupted and interrupted trials in Experiment 1. Although interruption led to slower RTs in both experiments, the cost of interruption did not extend to the level where it affected search accuracy in Experiment 2. Search accuracy was higher overall in Experiment 2, but RTs between experiments were similar. This suggests that our critical result is not likely due to a speed-accuracy trade-off where participants responded more quickly and were less accurate in Experiment 1. Taken together, the current data suggest that people will make more errors when interrupted while engaged in a primary task that strongly engages our limited WM resources.

In Williams and Drew (2017) and our Experiment 2, we argue that the primary task did not rely heavily on WM because search consisted of looking for repeated targets. In both studies, interruption led to a time cost but not an accuracy cost. In the Williams and Drew study, eye tracking measures suggested that the time cost was driven by poor memory of previously examined regions of the lungs following an interruption. Although we did not track eye movements in Experiment 2, this prior work suggests that poor memory for what areas were previously searched may have driven the increase in time associated with interruptions here as well. This raises the possibility that in situations where target representation is held in LTM, WM resources may be better utilized to keep track of areas that have been examined in the search array because this spatial information will not have to compete with target information (e.g., target features) for WM resources. Consistent with this possibility, research has shown that WM is recruited to maintain visual and spatial information concurrently (Berggren & Eimer, 2019; Oh & Kim, 2004; Woodman & Luck, 2004).

It may seem that the lack of an accuracy cost in the Williams and Drew study and in our Experiment 2 challenges the link between interruption and errors that past research has established (Altmann et al., 2014; Li et al., 2008; Monk et al., 2008; Trafton et al., 2003; Trafton et al., 2011; Zish et al., 2015). However, this relationship has primarily been established during procedural tasks with multiple steps (generally multiple goals) that must be held in WM. In this context, errors made upon resuming a task are the result of interruptions disrupting the memory which corresponds to the recently completed action. In comparison, typical visual search tasks are defined by a single task (search for targets) in which errors do not occur at a particular step. Rather than working through a sequence of different problems that require unique responses, such as in the UNRAVEL task, visual search involves a continuous process of scanning objects within visual arrays and comparing those objects to a target representation that is held in mind in order to determine whether a target is present or absent. When the target is the same through the course of an experiment and the memory demands on target representation are relatively low, such as in the Williams and Drew study and our Experiment 2, we argue that this visual search task does not place as high demand on WM as sequential tasks such as UNRAVEL. From this perspective, it may not be surprising that our group has consistently observed no cost on primary task accuracy in response to interruptions when the target is consistent across trials.

Our findings suggest that the cost of interruption is modulated by the level to which a task engages WM, which fits in well with investigations that have demonstrated that the cost of interruption is also influenced by individual differences in WM capacity (Drews & Musters, 2015; Foroughi et al., 2016; Meys & Sanderson, 2013; Werner et al., 2011). This research suggests that WM plays a critical role in determining the magnitude of the interruption cost through multiple avenues. However, prior research has focused on how individual differences in WM need to be considered when designing solutions that mitigate the negative effects of interruptions. In some cases, researchers speculate that job selection or job assignment criteria could take into account whether an individual’s WM capacity shields an individual from the negative effects of interruptions (Drews & Musters, 2015; Werner et al., 2011). Along these lines, researchers have also raised the possibility that informing individuals of their strengths and weaknesses will enable them to better manage their workload in the face of interruptions (Meys & Sanderson, 2013). Though individual differences in WM capacity is an important factor to consider, our study highlights the need to consider the extent to which the task itself engages WM.

Our data suggest that across the population, people will make more mistakes when they are interrupted during a task that places a heavy demand on WM. In a medical setting, such as diagnostic radiology, although an accurate and timely diagnosis are both important outcomes, diagnostic accuracy ultimately determines whether life-threatening conditions such as cancer are detected or overlooked. To the extent that the effects of interruptions found in our study generalize to radiologists, our results imply that radiologists are more likely to make an incorrect diagnosis when their search through medical images places a demand on WM. Therefore, our research suggests that task design plays a crucial role in determining when interruptions lead to large decrements in human performance. Thus, a viable direction for future research might be to identify the ways in which workflow environments can be modified such that they limit the disruptive effect of interruptions.

Conducting studies online through platforms, such as Prolific, provides the opportunity to collect a large sample of data efficiently. For the current study, we collected data sets on 62 subjects in less than a month. In comparison, it took approximately four months to collect data on 59 subjects for the pilot study that preceded this work. Overall, the results from the current online study are similar to the results from the pilot study that was conducted in our laboratory. We are excited to conduct additional interruption studies in order to examine how the timing or the frequency of interruptions influences the disruptive effects of interruption. The turnaround time to conduct these future studies will be significantly reduced by posting the new studies online. One of the concerns in conducting online studies is that many data sets will be discarded because participants’ computers are not optimized for cognitive science experiments where the precise timing of stimuli and events is critical. However, we implemented strict criteria to determine if a participant’s computer was too slow and would be unreliable for stimulus presentation (see participants section for the criteria). We believe this played an important role in decreasing the number of unreliable data sets that we collected. Thus, we hope that the current set of experiments will encourage fellow researchers to consider running their own interruption studies online.

### Limitations

A limitation of our work is that our experiments did not utilize complex medical images as stimuli. Rather, undergraduates were infrequently interrupted during search for relatively simple real-world objects within artificial displays. Therefore, more work is needed to determine whether the cost of interruption during visual search also generalizes to radiologists. Moreover, future work should utilize tasks that are more analogous to the type of search tasks that radiologists are required to complete. When doing so, these tasks (i.e., medical evaluations) should vary in the demands they place on memory since the current findings suggest that the cost of interruption is influenced by the WM demands associated with the primary task. For example, our findings suggest that a radiology task where there is single, well-understood goal, such as breast cancer screening, may be less susceptible to diagnostic accuracy costs in response to interruption than tasks that may require shuttling more information in and out of WM, such as initial evaluation of patients who arrive at the Emergency Room. This work should also take into account the high frequency in which radiologists are interrupted (Yu et al., 2014). This is an important factor to consider because prior research suggests that the frequency of interruptions plays an important role in determining the level to which a task is disrupted (Monk 2004).

Another limitation of this study is that we conceptualized our task manipulations in terms of their demands on WM, but these manipulations could also be described in terms of the precision of target representation and without invoking WM. More specifically, repeated search for the same target (Experiment 2) provides the opportunity to form—with greater detail—a mental representation of the target object. However, searching for targets that vary (Experiment 1) will likely limit the extent to which a precise target representation is generated. Research has shown that increases in template precision leads to a more efficient visual search (Bravo & Farid, 2009; Hout & Goldinger, 2015; Malcom & Henderson, 2009; Menneer et al., 2009; Schmidt & Zelinsky, 2009; Goldstein & Beck, 2018; Lavelle et al., 2021). Therefore, if search for repeated targets facilitates improvements in template precision, one would expect to see faster RTs over the course of target repetitions. Indeed, RTs are faster (Carlisle et al., 2011; Drew et al., 2018; Reinhart et al., 2016) and target detection is more accurate (Drew et al., 2018; Williams & Drew, 2018) when targets are consistent across runs of trials compared to when they vary. Given these findings, a more precise target template may also mitigate the performance costs of interruption. One possibility is that a high-fidelity representation of a target is less susceptible to the disruptive effects of interruption. Therefore, we cannot rule out that the cost of interruption was influenced by differences in the precision of target templates between Experiment 1 and 2. Although there is strong neurophysiological data that indicates that WM is more active when the search target changes, at present, it is unclear whether the driving force behind the observed effects is the precision of the target representation rather than WM engagement per se. Thus, further work is needed in order to better isolate the role of WM in modulating the cost of interruption. Nonetheless, we provide evidence that the design of a primary task can determine the type of performance costs associated with interruption.

### Conclusion

Identifying the factors that lead to medical errors requires an understanding of the limits of human performance and the cognitive consequences of environments that health practitioners operate within. Observational studies in medical settings and experimental studies in cognitive science provide ample evidence that interruptions create a context in which errors are made. In this study, we demonstrate that the type of task people complete is crucial for determining the level to which performance is disrupted by interruptions. The current findings provide strong evidence that the cost of interruption is more severe when people perform a search task that asks them to find a new target on each trial compared to the same target. Thus, the value of finding ways to decrease the number of interruptions likely depends heavily on the nature of the clinician’s primary task. Our data suggest that more complex search tasks that require greater engagement of working memory to represent the search target are more likely to lead to increased search errors. Thus, an implication of this research is that interruptions may be less disruptive when a medical evaluation involves a search task that requires a radiologist to screen for a well-defined abnormality compared to when there is a large number of potential findings. Although we readily acknowledge that radiologists searching patient cases for abnormalities is very different than undergraduates searching for pre-specified objects, the underlying premise of this research is that both populations use the same underlying search mechanisms (Wolfe et al., 2016). Ultimately, we believe that this sort of basic research, which investigates the factors that play a role in determining the cost of interruption, is an important starting point for guiding future research with these expert populations.

## Data Availability

All of the relevant data, experiment code and analysis scripts are published on the OSF website. Additional data and scripts are available from the corresponding author upon request. https://osf.io/4v3kp.
